# Lifelong reduction in complex IV induces tissue‐specific metabolic effects but does not reduce lifespan or healthspan in mice

**DOI:** 10.1111/acel.12769

**Published:** 2018-04-25

**Authors:** Sathyaseelan S. Deepa, Gavin Pharaoh, Michael Kinter, Vivian Diaz, Wilson C. Fok, Kaitlyn Riddle, Daniel Pulliam, Shauna Hill, Kathleen E. Fischer, Vanessa Soto, Constantin Georgescu, Jonathan D. Wren, Carlo Viscomi, Arlan Richardson, Holly Van Remmen

**Affiliations:** ^1^ Aging and Metabolism Research Program Oklahoma Medical Research Foundation Oklahoma City Oklahoma; ^2^ Department of Physiology University of Oklahoma Health Sciences Center Oklahoma City Oklahoma; ^3^ Department of Cellular and Structural Biology Barshop Institute for Longevity and Aging Studies University of Texas Health Science Center San Antonio San Antonio Texas; ^4^ Division of Hematology Department of Medicine Washington University in St. Louis St. Louis Missouri; ^5^ Biology Department University of Alabama at Birmingham Birmingham Alabama; ^6^ Arthritis & Clinical Immunology Research Program Division of Genomics and Data Sciences Oklahoma Medical Research Foundation Oklahoma City Oklahoma; ^7^ MRC‐Mitochondrial Biology Unit University of Cambridge Cambridge UK; ^8^ Department of Geriatric Medicine Reynolds Oklahoma Center on Aging University of Oklahoma Health Sciences Center Oklahoma City Oklahoma; ^9^ Oklahoma City VA Medical Center Oklahoma City Oklahoma

**Keywords:** cytochrome *c* oxidase, dietary restriction, lifespan, mitochondria, SURF1, mitochondrial unfolded protein response

## Abstract

Loss of SURF1, a Complex IV assembly protein, was reported to increase lifespan in mice despite dramatically lower cytochrome oxidase (COX) activity. Consistent with this, our previous studies found advantageous changes in metabolism (reduced adiposity, increased insulin sensitivity, and mitochondrial biogenesis) in *Surf1* ^−/−^ mice. The lack of deleterious phenotypes in *Surf1* ^−/−^ mice is contrary to the hypothesis that mitochondrial dysfunction contributes to aging. We found only a modest (nonsignificant) extension of lifespan (7% median, 16% maximum) and no change in healthspan indices in *Surf1* ^−/−^ vs. *Surf1* ^+/+^ mice despite substantial decreases in COX activity (22%–87% across tissues). Dietary restriction (DR) increased median lifespan in both *Surf1* ^+/+^ and *Surf1* ^−/−^ mice (36% and 19%, respectively). We measured gene expression, metabolites, and targeted expression of key metabolic proteins in adipose tissue, liver, and brain in *Surf1* ^*+/+*^ and *Surf1* ^−/−^ mice. Gene expression was differentially regulated in a tissue‐specific manner. Many proteins and metabolites are downregulated in *Surf1* ^−/−^ adipose tissue and reversed by DR, while in brain, most metabolites that changed were elevated in *Surf1* ^−/−^ mice. Finally, mitochondrial unfolded protein response (UPR^mt^)‐associated proteins were not uniformly altered by age or genotype, suggesting the UPR^mt^ is not a key player in aging or in response to reduced COX activity. While the changes in gene expression and metabolism may represent compensatory responses to mitochondrial stress, the important outcome of this study is that lifespan and healthspan are not compromised in *Surf1* ^−/−^ mice, suggesting that not all mitochondrial deficiencies are a critical determinant of lifespan.

## 1 INTRODUCTION

It has been proposed that compromised mitochondrial function is a causal determinant of aging and age‐related disease. While this is a reasonable assumption, the data to support a universal direct link between reduced mitochondrial function and lifespan surprisingly do not exist. For example, we have previously shown that heterozygous (*Sod2* ^+/−^) mice with reduced levels of MnSOD, a mitochondrial antioxidant enzyme, have compromised mitochondrial function but no reduction in lifespan (Mansouri et al., 2006; Van Remmen et al., 2003; Williams et al., 1998). In contrast, a study by Dell'agnello et al. (2007) reported that mice lacking expression of the Surf1 gene, which codes for the SURF1 assembly protein for complex IV (cytochrome *c* oxidase, COX) of the electron transport chain (Zhu et al., 1998), show a significant increase in mean lifespan despite a dramatic reduction in COX activity (Dell'agnello et al., 2007). These studies support the concept that mitochondrial function and lifespan are not causally linked.

In humans, the SURF1 mutation leads to COX deficiency (>90%) in multiple tissues and causes a fatal neurological disorder, Leigh syndrome (Tiranti et al., 1998). Mice lacking active SURF1 protein (*Surf1* ^−/−^ mice) were developed to study Leigh Syndrome (Dell'agnello et al., 2007). However, despite reduced COX activity in all tissues, the *Surf1* ^−/−^ mice exhibited an unexpected increase in lifespan and a number of phenotypes that do not seem consistent with reduced mitochondrial ETC activity. For example, *Surf1* ^−/−^ mice are protected from kainic acid‐induced neuronal damage, and isolated neurons from *Surf1* ^−/−^ mice are resistant to cytotoxic effects of glutamate (Dell'agnello et al., 2007). Studies from our laboratory have shown that *Surf1* ^−/−^ mice have reduced fat mass, improved insulin sensitivity, and enhanced memory, compared to wild‐type (*Surf1* ^+/+^) mice (Deepa et al., 2013; Lin et al., 2013). In addition, tissues from *Surf1* ^−/−^ mice show increased mitochondrial biogenesis and induction of the mitochondrial unfolded protein response (UPRmt), an evolutionarily conserved stress response pathway (Baker, Tatsuta & Langer, 2011). Fibroblasts from *Surf1* ^−/−^ mice also show increased resistance to cellular stresses (Pharaoh et al., 2016; Pulliam et al., 2014) suggesting that reduced COX activity induces compensatory mitochondrial stress response pathways.

To better understand how the loss of a key mitochondrial protein and dramatically reduced ETC Complex IV activity can generate the positive phenotypes listed above, we set out to critically assess the effect of *Surf1* deletion on lifespan in mice and to determine the physiological changes in response to reduced mitochondrial ETC that may contribute to increased lifespan. We repeated the lifespan analysis because (i) the original lifespan study was not intended to be an aging study and did not measure maximum lifespan in all cohorts in the study; (ii) the *Surf1* ^+/+^ mice had a short lifespan relative to previously reported values for this strain (B6D2F1); and (iii) because dietary restriction (DR) has been shown to increase lifespan, we were interested in testing whether DR would further increase lifespan in the *Surf1* ^−/−^ mice. Our data confirm that the *Surf1* ^−/−^ mice are able to avoid a reduction in lifespan despite significant loss of COX activity. These data provide evidence that reduced mitochondrial ETC activity is not a de facto negative determinant of lifespan, and one cannot assume that all deficiencies in mitochondrial proteins will translate to negative effects on lifespan.

## 2 RESULTS

### 2.1 COX activity is reduced in *Surf1* ^−/−^ mice

**Figure 1:**
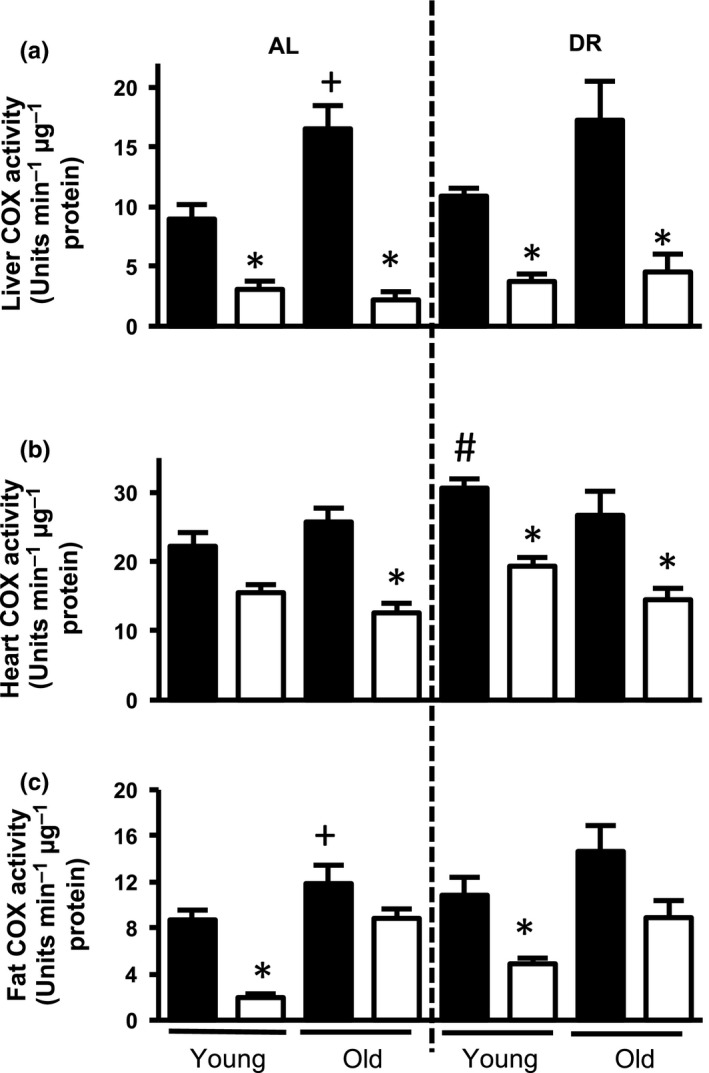
COX activity is reduced in *Surf1* ^−/−^ mice fed AL or a DR diet. COX activity in tissue homogenates of liver (a), heart (b), and WAT (c) of *Surf1* ^+/+^ (black bars) and *Surf1* ^−/−^ mice (white bars) fed AL or DR diet. Error bars represent mean ± *SEM* (*n* = 4–7). * *Surf1* ^+/+^ ‐AL vs. *Surf1* ^−/−^ ‐AL, + young vs. old; #AL vs. DR. */+/# *p* < .05

*Surf1* deficiency was previously reported to lead to a reduction in COX activity, without affecting activity of other ETC complexes (Dell'agnello et al., 2007; Pulliam et al., 2014). Here, we measured COX activity in liver, heart, and white adipose tissue (WAT) of young (8–12 months) and old (24–27 months) *Surf1* ^−/−^ and *Surf1* ^*+/+*^ female mice using tissue homogenates. COX activity is reduced in all three tissues ranging from 22% to 87% in young and old *Surf1* ^−/−^ mice, compared to *Surf1* ^*+/+*^ mice on either an AL or DR diet (Figure 1 and Table 1).

**Table 1:** COX activity is reduced in *Surf1* ^−/−^ mice fed AL or DR diet. COX activity from Figure 1 represented as percentage decrease in *Surf1* ^−/−^ mice compared to *Surf1* ^*+/+*^ mice

	% Decrease in *Surf1* ^−/−^ mice compared to *Surf1* ^*+/+*^ mice			
	AL		DR	
	Young	Old	Young	Old
Liver	65.6 ± 14.6	86.7 ± 27.1	65.6 ± 10.9	73.7 ± 24.2
Heart	30.3 ± 2.2	51.0 ± 5.6	36.8 ± 2.4	45.7 ± 5.2
Fat	77.4 ± 12.6	25.4 ± 2.4	54.9 ± 5.6	39.2 ± 6.5

### 2.2 *Surf1* ^−/−^ mice lifespan and healthspan

**Figure 2:**
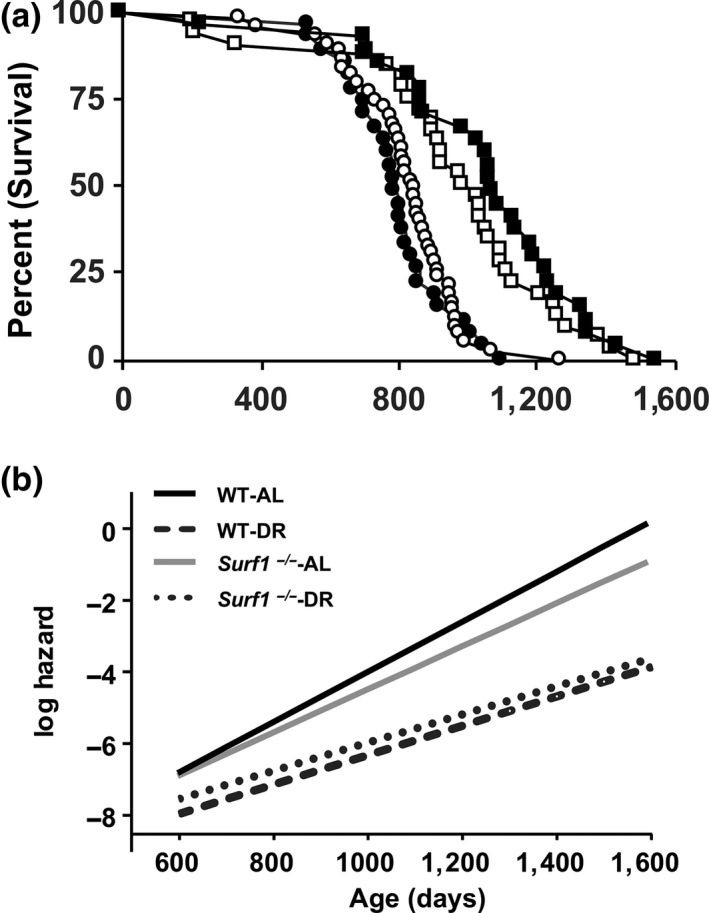
*Surf1* ^−/−^ mice have increased median lifespan when fed AL, and DR reduced median lifespan of *Surf1* ^−/−^ mice. (a) The Kaplan–Meier survival curves for *Surf1* ^+/+^and *Surf1* ^−/−^ female mice fed AL and DR diet. The curves represent *Surf1* ^+/+^ mice fed AL (black circle, *n* = 29), *Surf1* ^−/−^ mice fed AL (white circle, *n* = 32), *Surf1* ^+/+^ mice fed a DR diet (black square, *n* = 45), *Surf1* ^−/−^ mice fed a DR diet (white square, *n* = 44). (b) Gompertz plot for *Surf1* ^+/+^and *Surf1* ^−/−^ female mice fed AL and DR diet

We measured lifespan in *Surf1* ^*+/+*^ and *Surf1* ^−/−^ female mice fed an AL or 40% DR diet (Figure 2a, Table 2). *Surf1* ^−/−^ mice fed AL showed a marginal increase in median lifespan (6.6%, *p* = .05) as well as nonsignificant increases in mean (3.5%, *p* = .46) and maximal lifespan (15.9% *p* = .72) compared to *Surf1* ^+/+^ mice fed AL. DR robustly extended lifespan of both *Surf1* ^+/+^ and *Surf1* ^−/−^ mice, although to a lesser extent in *Surf1* ^−/−^ mice. Analysis of survival curves using the Kaplan–Meier log‐rank test showed no significant difference in the survival of *Surf1* ^−/−^ mice vs. *Surf1* ^+/+^ mice fed AL (*p* = .44) or a DR diet (*p* = .27), and confirmed the significant effect of the DR diet on survival. Gompertz analysis of survival is shown in Figure 2b. The Gompertz plot shows the log hazard function for each curve, modeled as a linear function of age. Both genotypes fed a DR diet showed a lower line slope, suggesting a slower age acceleration in response to DR. However, no difference was observed between *Surf1* ^+/+^ and *Surf1* ^−/−^ mice on either diet. Although both Kaplan–Meier log‐rank test and Gompertz analysis failed to show a statistically significant change in lifespan between *Surf1* ^*+/+*^ and *Surf1* ^−/−^ mice, we did detect small differences when we analyzed the difference in quartile survival time, between pairs of curves, for all quartiles (Q). Interestingly, *Surf1* ^−/−^ mice fed AL showed a significant increase in lifespan at Q.5 and Q.7, compared to *Surf1* ^+/+^ mice fed AL and *Surf1* ^−/−^ mice fed a DR diet showed a significant decrease in lifespan at Q.4 (*p* = 1.5 × 10^−2^) compared to *Surf1* ^+/+^ mice fed a DR diet (Table S1). Our findings show that deficiency of *Surf1* does not affect overall lifespan.

**Table 2:** Lifespan analysis of *Surf1* ^*+/+*^ and *Surf1* ^−/−^ mice fed AL or DR diet. The survival data from Figure 2 are expressed in days. Mean, median, 90% and maximum lifespan of *Surf1* ^−/−^ mice are compared to *Surf1* ^+/+^ mice in AL and DR groups

	*Surf1* ^*+/+*^ ‐AL	*Surf1* ^−/−^ ‐AL	*Surf1* ^*+/+*^ ‐DR	*Surf1* ^−/−^ ‐DR
Mean ± *SEM*	797 ± 27.7	825 ± 21.5	1063 ± 53.8	973 ± 53.6
Median	794	850^a^	1,077	1,009
90%	999	972	1,352	1,288
Maximum	1,098	1,273	1,600	1,486

a Indicates significant difference between genotypes.

**Figure 3:**
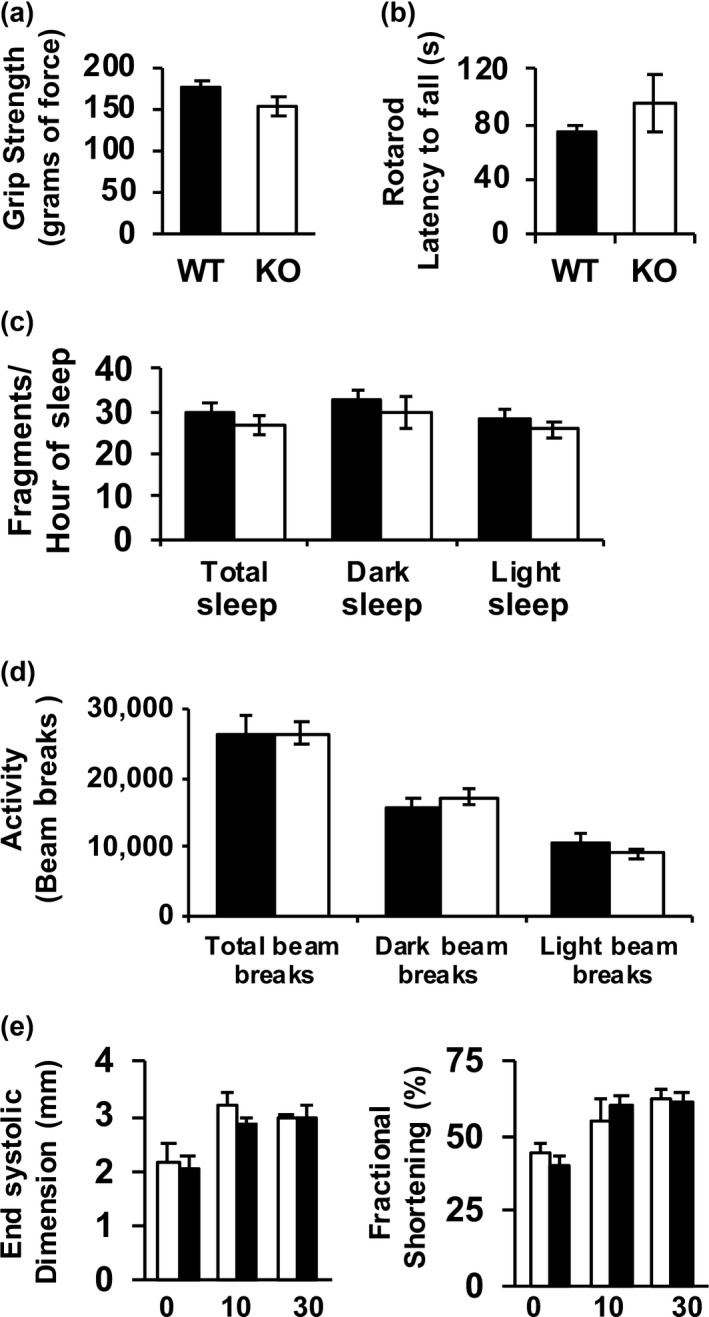
*Surf1* ^−/−^ mice do not show changes in markers of healthspan. (a) Grip strength measured in 28‐month‐old *Surf1* ^+/+^and *Surf1* ^−/−^ female fed AL. The grip force (in grams) was measured over 10 trials and the maximum force was recorded. (b) Rotarod performance measurement in 28‐month‐old *Surf1* ^+/+^ (black bars) and *Surf1* ^−/−^ (white bars) female fed AL. The average latency to fall from the rod (in seconds) was recorded. (c) Sleep fragmentation in 28‐month‐old *Surf1* ^+/+^ (black bars) and *Surf1* ^−/−^ (white bars) female fed AL expressed as the number of sleep bouts per hour of sleep (any period of inactivity (no beam breaks) greater than or equal to 40 s). (d) Spontaneous activity measured in AL‐fed 28‐month‐old *Surf1* ^+/+^ (black bars) and *Surf1* ^−/−^ (white bars) mice measured as the number of beam breaks (e) Cardiovascular functions end systolic dimension (left panel) and fractional shortening (right panel) in 20‐month‐old *Surf1* ^+/+^ (black bars) and *Surf1* ^−/−^ (white bars) female mice measured as previously described (Pulliam et al., 2014). Error bars represent mean ± *SEM*

Lifespan is a valuable measure of the ultimate cumulative effects of aging (Fischer et al., 2015). However, it has become increasingly evident that changes in markers that indicate the overall general health and vitality of an organism, that is healthspan, are also highly informative (Richardson et al., 2016). To assess the effects of complex IV deficiency in the *Surf1* ^−/−^ mice on healthspan, we measured several physiologic parameters including grip strength, rotarod performance, sleep/wake patterns, spontaneous cage activity in 28‐month‐old female mice and cardiovascular function in 20‐month‐old female mice. We found no significant changes in any of these parameters between *Surf1* ^*+/+*^ and *Surf1* ^−/−^ mice (Figure 3).

### 2.3 *Surf1* ^−/−^ mice fed AL but not a DR diet have a reduction in body weight and fat mass with age

**Figure 4:**
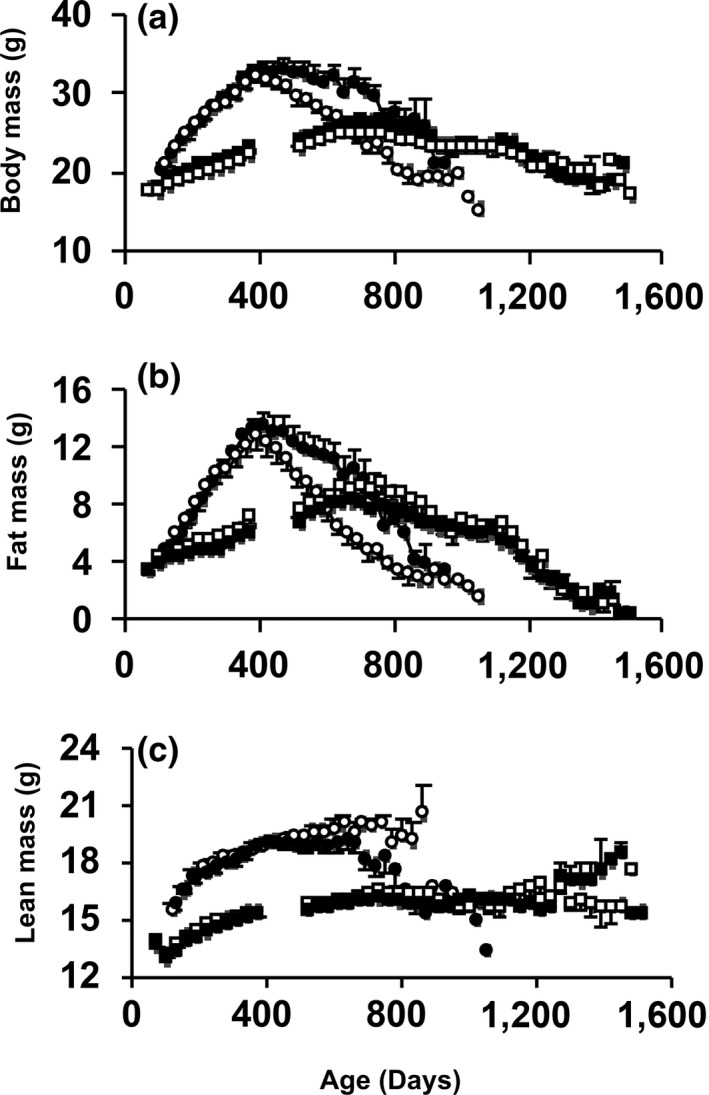
*Surf1* ^−/−^ mice fed AL, not a DR diet, have a reduction in body weight and fat mass with age. Change in body weight (a), fat mass (b), and lean mass (c) with age in *Surf1* ^+/+^ and *Surf1* ^−/−^ mice in the survival cohorts. The curves represent *Surf1* ^+/+^ mice fed AL (black circle), *Surf1* ^−/−^ mice fed AL (white circle), *Surf1* ^+/+^ mice fed a DR diet (black square), *Surf1* ^−/−^ mice fed a DR diet (white square). Error bars represent mean ± *SEM*

Previously, we reported reduced fat mass and body weight in young *Surf1* ^−/−^ male mice compared to *Surf1* ^*+/+*^ mice (Deepa et al., 2013). We found a similar pattern in loss of fat mass in this study. At 800 days of age, *Surf1* ^−/−^ mice have 25% reduction in body weight compared to age‐matched *Surf1* ^*+/+*^ mice (Figure 4a). In mice fed DR, *Surf1* ^*+/+*^ and *Surf1* ^−/−^ mice lost fat mass in a similar pattern (Figure 4b). Both genotypes on a DR diet showed similar changes in lean mass with age, however, at 800 days of age, *Surf1* ^−/−^ mice fed AL have a 15% lower lean mass than age‐matched *Surf1* ^*+/+*^ mice fed AL (Figure 4c).

### 2.4 Tissue‐specific differential regulation of pathways and genes in *Surf1* ^−/−^ mice

**Figure 5:**
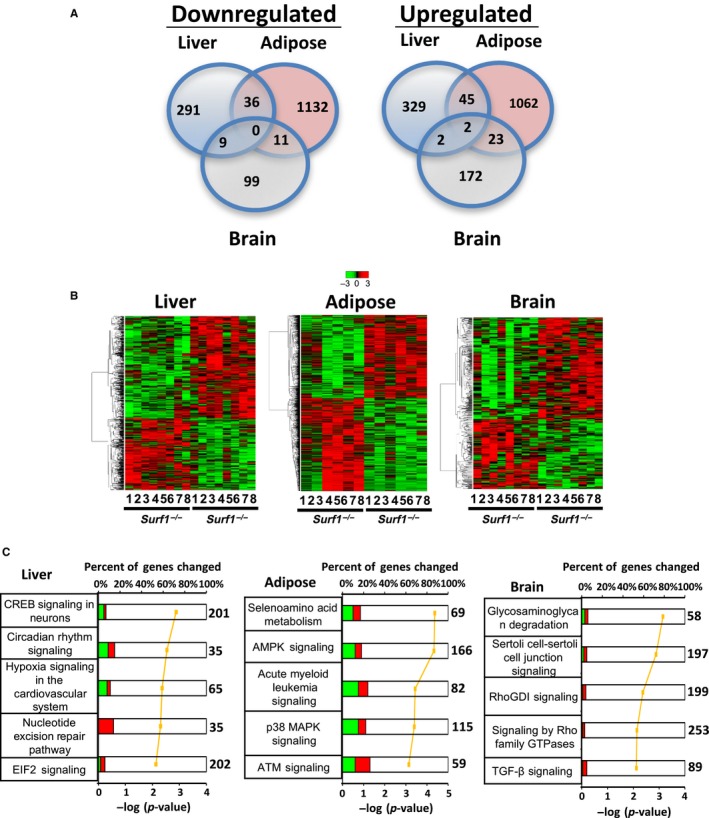
Tissue‐specific differential expression of genes and metabolic pathways in *Surf1* ^+/+^ and *Surf1* ^−/−^ mice. (a) Venn diagram showing number of genes significantly downregulated (left panel) or upregulated (right panel) in liver (blue), WAT (yellow), and brain (green) using a filtering criteria of *p* < .05. (b) Heatmap showing expression of all the significantly changed probes in liver (left panel), WAT (middle panel), and brain (right panel). The *Surf1* ^+/+^ and *Surf1* ^−/−^ samples were clustered using average linkage hierarchical cluster with Euclidean distance using a criteria of *p *<* *.05. Green represents downregulated genes and red represents upregulated genes. (c) The top five pathways from Ingenuity Pathway Analysis ranked by the lowest *p*‐values as determined by the Fisher's exact test are shown for liver (left panel), WAT (middle panel), and brain (right panel). The yellow line represents the ‐log of the *p*‐value and the bolded number on the right side of the graph represents the total number of possible genes of that pathway. Green color indicates percentage of downregulated genes, red color indicates percentage of upregulated genes, and white color indicates the percentage not found in the significant gene list. Table S2 lists the genes identified by Ingenuity Pathway Analysis

Microarray analysis was performed as an unbiased approach to compare gene expression of liver, WAT, and brain (cortex) of *Surf1* ^*+/+*^ and *Surf1* ^−/−^ mice fed AL. We used a *p* < .05 cutoff to detect expression of genes significantly altered by *Surf1* deficiency. In liver from *Surf1* ^−/−^ mice, 714 transcripts showed a significant change compared to *Surf1* ^*+/+*^ mice: 336 genes were downregulated and 378 genes were upregulated (Figure 5a). In WAT of *Surf1* ^−/−^ mice, 2,311 transcripts showed significant changes compared to *Surf1* ^+/+^ mice: 1,179 genes were downregulated and 1,132 genes were upregulated. In brain from *Surf1* ^−/−^ mice, 318 transcripts showed significant changes compared to *Surf1* ^+/+^ mice, 119 genes were downregulated and 199 genes were upregulated. However, none of the downregulated genes were the same among the three tissues studied. In contrast, among the upregulated transcripts, ADAM Metallopeptidase Domain 11 (Adam11) and Transmembrane and Coiled‐Coil Domains 3 (Tmco3) were upregulated in all three tissues. Transcripts in liver and WAT of *Surf1* ^−/−^ mice showed an overlap: 36 of the downregulated genes and 45 of the upregulated genes were similar in liver and WAT. Similarly, 11 of the downregulated genes and 23 of the upregulated genes in *Surf1* ^−/−^ mice were similar in WAT and brain. Comparison of transcripts in brain and liver showed that nine of the genes are downregulated and two of the genes upregulated in both brain and liver (Figure 5a).

Heatmap analysis of genes that showed a significant change compared to *Surf1* ^*+/+*^ mice is shown in Figure 5b. Using ingenuity pathway analysis (IPA), we found that in liver, WAT, and brain 21, 95, and 30 pathways, respectively, are altered due to *Surf1* deficiency. Figure 5c shows the top 5 canonical pathways that are changed in liver, WAT, and brain of *Surf1* ^−/−^ mice. Even though we found significant changes in pathways in liver, WAT, and brain of *Surf1* ^−/−^ mice, there was no overlap between the pathways in the three tissues. This suggests that *Surf1* deficiency has a differential effect on metabolism/signaling pathways in different tissues. Table S2 shows the genes upregulated or downregulated in liver, WAT, and brain of *Surf1* ^−/−^ mice.

### 2.5 Effect of diet and genotype on tissue metabolites in *Surf1* ^−/−^ mice

A total of 221 metabolites were analyzed in adipose tissue of *Surf1* ^*+/+*^ and *Surf1* ^−/−^ mice and of these 57 metabolites were increased and 46 metabolites were decreased (Table S3). *Surf1* deficiency altered the level of 28 metabolites in adipose tissue in mice fed an AL diet: two were increased and the other 26 were decreased. Metabolites that change in response to *Surf1* deficiency belong to the following metabolic pathways: amino acid (11 decreased); carbohydrate (two increased); lipid (nine decreased and one increased); and nucleotide (four decreased and one increased) (Table S3). In adipose tissue from *Surf1* ^+/+^ mice, 22 metabolites changed (eight increased and 14 decreased) and in *Surf1* ^−/−^ mice 42 metabolites showed a significant change (39 increased and three decreased). Thus, *Surf1* ^−/−^ mice showed a greater effect on metabolites in response to DR and the greatest effect was observed in metabolites in the lipid metabolism pathway (Table S3).

The liver tissue dataset is comprised of a total of 327 biochemicals of which 47 showed a significant change (39 increased and eight decreased) (Table S4). Several amino acids, including phenylalanine, glycine, tryptophan, leucine, and valine, were present at higher levels in *Surf1* ^−/−^ mice livers, suggesting higher levels of protein turnover in *Surf1* ^−/−^ mice livers. A striking accumulation of medium‐chain free fatty acids—12:0, 10:0, 9:0, and 8:0—as well as the acylglycine conjugate valerylglycine are symptomatic of a block in the function of the medium‐chain acyl‐CoA dehydrogenase (MCAD).

### 2.6 Metabolic pathway analysis in adipose tissue

**Figure 6:**
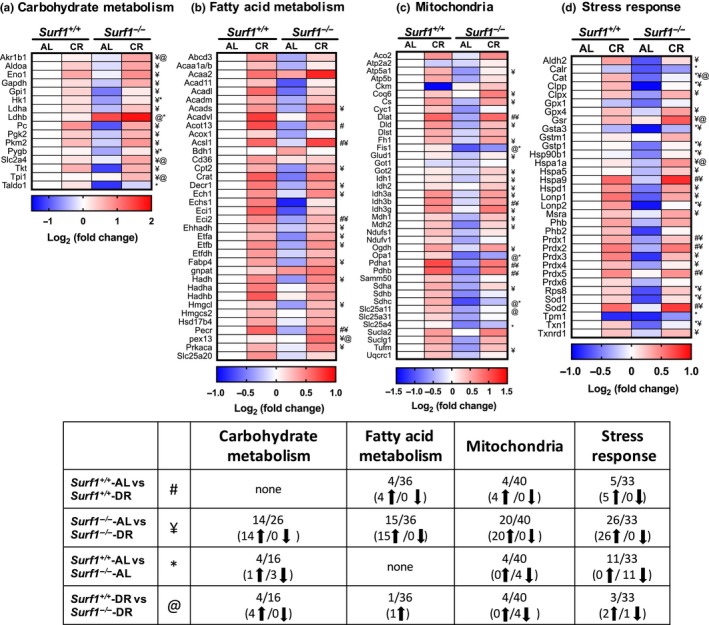
Metabolic pathways are altered in *Surf1* ^−/−^ mice adipose tissue and DR reverses most of these changes. Heatmaps representing changes in protein expression of enzymes/proteins in *Surf1* ^−/−^ mice compared to *Surf1* ^+/+^ mice fed AL or DR in carbohydrate metabolism (a), fatty acid metabolism (b), TCA cycle, ETC proteins, and assorted mitochondrial proteins (c), and stress response (d) in WAT obtained by targeted mass spectrometry analysis. Blue indicates downregulated proteins, white is unchanged, and red is upregulated. A summary of the data (e) represents number of enzymes/proteins in the pathway that are altered. Statistical significance determined by two‐Way ANOVA with Tukey's post hoc test (*n* = 7–8)

Previously, we found that deficiency of *Surf1* in male mice shift metabolism to increased fatty acid oxidation in peripheral tissues (Deepa et al., 2013). Here, we used a targeted quantitative proteomic approach to assess the changes in metabolic pathways in WAT. We analyzed WAT from *Surf1* ^−/−^ mice and *Surf1* ^*+/+*^ mice fed AL and DR diet less than 400 days of age to see changes prior to fat loss. Of the 16 proteins analyzed in carbohydrate metabolism, only four proteins showed a significant difference due to *Surf1* deficiency, three were downregulated (Hk1, Pygb, Taldo) and one was upregulated (Ldhb). DR did not alter the expression of any of the proteins in the carbohydrate metabolism panel in *Surf1* ^*+/+*^ mice compared to AL‐fed *Surf1* ^*+/+*^ mice, however, 14 proteins in *Surf1* ^−/−^ mice are upregulated due to DR, compared to AL‐fed *Surf1* ^−/−^ mice (Figure 6). DR also showed a genotype‐specific effect on protein expression: expression of Akr1b1, Ldhb, Slc2a4, and Taldo is increased in *Surf1* ^−/−^ mice fed DR diet, compared to *Surf1* ^+/+^ mice fed DR diet.

Protein expression analysis of a panel of 36 enzymes involved in mitochondrial and peroxisomal fatty acid oxidation pathways revealed no changes in *Surf1* ^−/−^ mice fed an AL diet (Figure 6). Four of the proteins in the fatty acid oxidation panel were upregulated in *Surf1* ^*+/+*^ mice and 15 are upregulated in *Surf1* ^−/−^ mice on DR. Pex13 is the only protein that showed a significant increase in DR‐fed *Surf1* ^−/−^ mice compared to DR‐fed *Surf1* ^*+/+*^ mice (Figure 6).

Analysis of a panel of 40 enzymes/proteins in tricarboxylic acid cycle (TCA), electron transport chain (ETC), and mitochondrial enzymes/proteins showed that protein expression of four proteins was downregulated by *Surf1* deficiency (Fis1, Opa1, Sdhc, and Slc25a4) (Figure 6). Twenty of the 40 proteins analyzed in the mitochondrial panel were upregulated in *Surf1* ^−/−^ mice on DR diet, whereas only four proteins were upregulated in *Surf1* ^*+/+*^ mice fed the DR diet (Figure 6). *Surf1* ^−/−^ mice fed DR diet showed downregulation of four proteins (Fis1, Opa1, Sdhc, and Slc25a11) compared to *Surf1* ^+/+^ mice fed DR diet. Therefore, Fis1, Opa1, and Sdhc were downregulated in *Surf1* ^−/−^ independent of diet.

Among antioxidants, chaperones, heat shock proteins, and proteases (a total of 33 proteins) in the stress response panel, absence of *Surf1* reduced the expression of 11 proteins in *Surf1* ^−/−^ mice compared to *Surf1* ^*+/+*^ mice (Figure 6). DR increased the expression of 25 proteins in *Surf1* ^−/−^ mice fed DR vs. AL diet, whereas only five proteins were elevated in *Surf1* ^+/+^ mice fed DR diet. DR also showed a genotype‐specific effect on protein expression in the mitochondrial panel: Gsr and Hspa1a were increased and catalase was decreased in *Surf1* ^−/−^ mice fed DR diet, compared to *Surf1* ^*+/+*^ mice fed DR diet (Figure 6, Table S5).

### 2.7 UPR^mt^‐associated proteins and mitochondrial ETC complex subunits are induced transcriptionally in *Surf1* ^−/−^ mice liver, but not in heart

Because the effect of aging on the UPR^mt^ has not been reported in mice, we measured transcript and protein levels of UPR^mt^‐associated proteins ClpP, LonP1, and Hsp60 in young and old *Surf1* ^−/−^ and *Surf1* ^+/+^ mice. In young *Surf1* ^−/−^ mice liver, transcript levels of ClpP, LonP1 are elevated by 1.4‐ and 2.9‐fold, respectively, compared to young *Surf1* ^*+/+*^ mice (Figure S1a). However, an induction in mRNA levels for UPR^mt^‐associated proteins is not observed in *Surf1* ^−/−^ mice heart (Figure S1b), suggesting a tissue‐specific effect of UPR^mt^ in response to *Surf1* deficiency. Our findings suggest that UPR^mt^‐associated proteins are induced only when the mice are young, not old, and they also show a tissue‐specific difference in their expression with age. The absence of UPR^mt^ induction, assessed by the transcript levels of UPR^mt^‐associated proteins, in old *Surf1* ^−/−^ mice suggests that the UPR^mt^ is not directly involved in lifespan extension. However, the effect of UPR^mt^ induction at young age on lifespan is not known.

*Surf1* ^−/−^ male mice are reported to have elevated mitochondrial biogenesis and increased expression of ETC subunits (Pulliam et al., 2014). Here, assessment of transcript levels of ETC subunits in liver of female *Surf1* ^−/−^ and *Surf1* ^*+/+*^ mice showed that transcript levels of ETC subunits NDUFS3 (3.8‐fold), ND1 (2.2‐fold), SDHA (3.9‐fold), SDHB (3.2‐fold), Reiske protein (3.5‐fold), COX2 (2.3‐fold), and ATPase 6 (1.6‐fold) were significantly elevated in young *Surf1* ^−/−^ mice (Figure S1e). However, there was no significant difference in the transcript levels of the ETC proteins in old *Surf1* ^−/−^ mice liver, compared to old *Surf1* ^+/+^ mice (Figure S1e). In contrast to the changes in ETC subunits in liver, ETC subunit mRNA levels were similar in *Surf1* ^+/+^and *Surf1* ^−/−^ mice heart at young and old ages (Figure S1f).

## 3 DISCUSSION

The key finding of this study is that a 22%–87% reduction in activity of a mitochondrial ETC complex (Complex IV) across all tissues measured does not significantly limit lifespan or negatively affect healthspan. Further, we show numerous and tissue‐specific changes in metabolism, protein, and gene expression in response to the loss of *Surf1*. For many years, there has been a widely held hypothesis that mitochondrial function decreases during aging and contributes to aging and age‐related physiologic decline. Specifically, the Mitochondrial Theory of Aging predicts that damage to the mitochondrial ETC and mitochondrial DNA over time accumulates and contributes to a spiral of compromised mitochondrial function that facilitates aging (Harman, 1972). The data we present here do not support this hypothesis. Rather, our results support the concept of a threshold effect for loss of mitochondrial function and provide clear evidence for a link between the ETC and metabolism.

Despite the commonly held concept that mitochondrial dysfunction is directly related to limited lifespan, the evidence available to support this is not unequivocal. For example, reduced ETC activity can lead to increased lifespan in *C. elegans* mitochondrial ETC (Mit) mutants (Munkácsy & Rea, 2014). Specifically, RNAi knock‐down of genes encoding subunits of ETC complexes I, III, or IV, and mitochondrial enzyme CoQ7 (Clk‐1) results in lifespan extension (Dillin et al., 2002; Lee et al., 2003; Munkácsy & Rea, 2014). Similar to the findings in *C. elegans*, inhibition of all ETC complexes, except complex II, extended lifespan in flies (Copeland et al., 2009). These studies in invertebrates are in clear contrast to the idea that mitochondrial function limits lifespan and the mechanisms responsible have not been defined. In mice, the effect of compromised mitochondrial ETC on lifespan is varied. Mice heterozygous for the *Mclk* gene, the homolog of the *C. elegans clk1* gene (the first reported long‐lived mitochondrial mutant in *C elegans*), have an extended lifespan (Lapointe, Stepanyan, Bigras & Hekimi, 2009), while Mclk homozygous null mice show a dramatically shortened lifespan (Hughes & Hekimi, 2011). Likewise, mice with a homozygous deletion of an ETC complex I subunit (Ndufs4 knockout mice) also show reduced longevity. Both of these mutant mouse models are very short‐lived and have severe respiratory and metabolic phenotypes (Kruse et al., 2008; Wang, Oxer & Hekimi, 2015). One explanation for the seemingly contradictory effects of ETC mutation and longevity is a threshold effect in which compromised mitochondrial function alters cellular function only once a critical deficiency in ATP production in reached. In the context of mitochondrial mutations that cause mitochondrial disease, the variable phenotypes that occur have been tied to a critical threshold of mutation load (approximately 80%–90%) above which the phenotypes associated with disease occur (Rossignol et al., 2003). For example, heterozygous knock‐in mice with reduced levels of DNA polymerase γ, a mitochondrial DNA proofreading enzyme, have a 30‐fold increase in mitochondrial DNA mutation load but show no deleterious phenotypes and have a normal lifespan while homozygous null mutants have a dramatic reduction in lifespan (Vermulst et al., 2007). Using cybrids containing a Complex I (ND5) mutation, Bai, Shakeley and Attardi (2000) showed that reduced mitochondrial respiration occurred only after the percentage of heteroplasmy reached 60%. Likewise, other studies have shown that inhibition of ETC complexes will not affect respiration or ATP production until a threshold is exceeded. In fact, this biochemical threshold was shown to be at 75% inhibition for COX in an in vitro analysis using isolated muscle mitochondria in vitro (Letellier, Heinrich, Malgat & Mazat, 1994).

Consistent with the threshold concept, we tested mitochondrial function in the *Surf1* ^−/−^ mice using standard in vitro assays and found no loss of mitochondrial function and no evidence for increased ROS generation (Pulliam et al., 2014). It is possible that this is somehow related to an increase in mitochondrial biogenesis to compensate for the reduction in COX activity in tissues of the *Surf1* ^−/−^ mice and inhibit any pathophysiological consequences at the cellular level. However, there is evidence that the mitochondria in the *Surf1* ^−/−^ mice are impaired to some extent in vivo. For example, the *Surf1* ^−/−^ mice have a significantly diminished treadmill endurance capacity and elevated blood lactate, consistent with impaired mitochondrial function (Pulliam et al., 2014).

The goal for this study was to determine the metabolic changes that occur in vivo in response to the reduction in SURF1 and Complex IV activity that might contribute to the increase in lifespan in *Surf1* ^−/−^ mice originally reported by Dell'agnello et al. (2007). We were also interested in whether DR could extend this lifespan extension. Thus, we repeated the lifespan study in female mice under AL and DR (40% restriction) conditions. However, our lifespan analysis revealed only a slight and nonsignificant increase (7%) in median lifespan, compared to the 17% increase previously reported for female *Surf1* ^−/−^ mice. Despite the discrepancy in the survival results, the critical fact remains that we did not see compromised lifespan despite the severe reduction in complex IV activity. This is not the first time a study was unable to replicate the increase in lifespan shown in an original study. For example, knockout mouse models for the signaling molecule p66Shc (p66shc^−/−^ mice) (Migliaccio et al., 1999), insulin receptor substrate 2 (Irs2) (Irs2^+/−^ mice) (Taguchi, Wartschow & White, 2007), and the IGF1 receptor (*Igf1r* ^*+/−*^ mice) (Holzenberger et al., 2003) all showed substantial lifespan extension in initial reports that was not confirmed in follow‐up studies (Bokov et al., 2011; Ladiges et al., 2009; Liang et al., 2003 *;* Ramsey et al., 2014; Selman, Lingard, Gems, Partridge & Withers, 2008; Selman et al., 2007; Unnikrishnan, Deepa, Herd & Richardson, 2017). Differences in genetic background can potentially modulate lifespan results. However, this was not a contributing factor in our results as the mice in our study and the study by Dell'agnello were on the same genetic background (B6D2F1/J). In contrast, a key difference between the lifespan results in the *Surf1* ^−/−^ mice lifespan study by Dell'agnello et al. (2007) and our study is the mean lifespan of the *Surf1* ^+/+^ mice. They reported a mean lifespan of 700 days for female *Surf1* ^+/+^ mice. This is considerably lower than previously reported for female *Surf1* ^+/+^ mice on B6D2F1/J background, that is, a mean lifespan 826 days (Yamate, Tajima, Kudow & Sannai, 1990). Hence, it is possible that the reduced lifespan of *Surf1* ^+/+^ mice contributed to the observed lifespan extension in *Surf1* ^−/−^ mice. The *Surf1* ^+/+^ mice in our cohort show a mean lifespan consistent with previously reported values (797 days), while the *Surf1* ^−/−^ mice in our cohort had a mean survival of 825 days. Interestingly, this is very close to the lifespan originally reported by Dell'agnello et al. (2007) (820 days), further supporting the possibility that the reduced *Surf1* ^+/+^ lifespan contributed to the lifespan extension they reported. We found that DR extended lifespan in both *Surf1* ^+/+^ and *Surf1* ^−/−^ mice; however, the effect was surprisingly less in the *Surf1* ^−/−^ mice (18% and 17% extension in mean and maximum lifespan, respectively, in the *Surf1* ^−/−^ mice vs. a 33% and 46% extension in *Surf1* ^+/+^ mice). Our metabolic and phenotypic data unfortunately do not reveal a plausible explanation for the reduced response of the *Surf1* ^−/−^ mice to DR, but one hypothesis might be that mitochondrial function may be more adversely affected by lower nutrient flux in response to DR in the mice with compromised COX activity.

Our previous studies in young adult male *Surf1* ^−/−^ mice showed interesting metabolic phenotypes such as reduced fat mass, increased insulin sensitivity, and increased mitochondrial biogenesis in some tissues. There was also an induction of the mitochondrial unfolded protein response (UPR^mt^) in several tissues, suggesting that the misassembly of Complex IV subunits due to the lack of the SURF1 assembly factor might induce upregulation of proteostasis and other protective mechanisms that may be related to the increase in lifespan. In the current study, we did not find a clear induction of the UPR^mt^. One possible reason for this is that our original studies were performed using male mice and the current study is in female mice. We also did not find a clear relationship between the UPR^mt^ and aging in this study. In addition, the most consistent finding in all the metabolic data we collected is the fact that COX deficiency affects gene expression metabolites and metabolic pathways in a tissue‐specific manner. Likewise, DR shows tissue‐specific effects on proteins and metabolites in *Surf1* ^+/+^ and *Surf1* ^−/−^ mice. In the current study, our metabolic studies mainly focused on adipose tissue, liver and, brain. Adipose tissue and liver play a role in metabolism, and the *Surf1* ^−/−^ mice tend to have less fat and improved insulin sensitivity (Deepa et al., 2013). We measured changes in brain, because many genetic manipulations restricted to brain have been shown to affect lifespan. For example, there is a report showing that a CNS‐restricted knockout of *Surf1* can extend lifespan in fruit flies (Zordan et al., 2006). Adipose tissue had by far the most changes in gene expression. Over 2,000 genes were differentially regulated in adipose tissue between *Surf1* ^+/+^ and *Surf1* ^−/−^ mice. These changes may be related to the dramatic loss of fat mass that occurs in the *Surf1* ^−/−^ mice over their lifespan. Metabolomic analysis in adipose tissue showed 28 of 221 metabolites were different between the *Surf1* ^+/+^ and knockout mice on AL diet but only half as many (11 metabolites) differed in *Surf1* ^+/+^ vs. *Surf1* ^−/−^ mice on DR. This may be related to the fact that there is no differential loss of fat mass in the *Surf1* ^−/−^ mice on DR and may also be related to the reduced lifespan extension in the *Surf1* ^−/−^ mice on DR compared to *Surf1* ^+/+^ mice on DR. In summary, the most important finding in this study is that reduced complex IV activity throughout the lifespan does not negatively impact lifespan or healthspan. The long held idea that mitochondrial dysfunction is a negative determinant of aging should be tempered by the idea that not all mitochondrial impairments do not accelerate aging or physiologic decline and may in fact have hidden beneficial effects on metabolism.

## 4 EXPERIMENTAL PROCEDURES

### 4.1 Animals and lifespan

*Surf1* ^−/−^ mice were generated as described by Dell'agnello et al. (2007). Experiments were conducted in female *Surf1* ^−/−^ B6D2F1/J (C57BL/6J_DBA/2) mice and control littermates (*Surf1* ^+/+^), unless otherwise stated, and were performed according to the protocol approved by the IACUC at the University of Texas Health Science at San Antonio. Mice for the lifespan study were generated by crossing *Surf1* ^*+/−*^ mice to each other to obtain *Surf1* ^−/−^ and *Surf1* ^+/+^ mice and only females were used for the study. For the lifespan study using AL‐fed mice, we used 32 *Surf1* ^−/−^ mice and 29 *Surf1* ^+/+^, and for the dietary restricted lifespan study, 44 *Surf1* ^−/−^ mice and 45 *Surf1* ^+/+^were used. Mice (5 animals/cage) were housed under barrier conditions using micro‐isolator cages and were fed AL a standard NIH‐31 chow diet (Harlan Teklad, Madison, WI, USA) on a 12‐h dark‐light cycle. Dietary restriction was initiated at 2 months of age by feeding DR mice 60% of the diet (by weight, 40% restriction) consumed by the mice fed AL. For the lifespan experiments for mice AL or a DR diet, the mice were allowed to live out their natural lifespan and lifespans for mice were determined by recording the ages of spontaneous death of the mice. The mean, median, 10% (the mean lifespan of the longest‐lived 10% of animals), and maximum (the age of death for the longest‐lived mouse in the cohort) lifespans were calculated from the survival data for each group. Body composition of the mice was determined monthly by quantitative magnetic resonance (QMR, Echo Medial Systems, Houston, TX, USA). Food consumption was measured for a one week period every two weeks for 3 months and monthly for 11 months in 19‐20 mice (four cages) per genotype. There was no difference in food consumption between the *Surf1* ^+/+^ and Surf1^−/−^ mice. Surf1^−/−^ mice consumed 3.1 g ± 0.1 g per day and Surf1^+/+^ mice consumed 3.0 ± 0.0 g per day).

### 4.2 COX activity assay

COX activity was measured in liver, heart, and WAT homogenates as described (Spinazzi, Casarin, Pertegato, Salviati & Angelini, 2012). The rate of oxidation of cytochrome *c* was calculated from the change in absorbance at 550 nm. The extinction coefficient of 21.84/mM/cm was used to determine the enzymatic activity.

### 4.3 Microarray analysis

Microarray processing was carried out at the National Institute on Aging, Gene Expression and Genomics Unit as previously described (Fok et al., 2014).

### 4.4 Metabolomics

Frozen brain tissue (*n* = 8) and WAT (*n* = 8) were analyzed for metabolite levels by Metabolon (Durham, NC, USA). At the time of analysis, samples were extracted and prepared for analysis using Metabolon's standard solvent extraction method as previously described (Ghazalpour et al., 2014). Data were normalized by Metabolon and statistical analysis was performed using ANOVA with pairwise comparisons. False discovery analysis was then applied to the dataset using r package “*q* value” (v 1.30.0, Dabney, A and Storey, J). Metabolon imputed the data for metabolites that had samples that were not detected using the lowest detected data value available for that metabolite. We excluded all metabolites that had imputed value >20% in the groups we analyzed for the pairwise comparisons. Statistical significance was indicated at *q* value < 0.05 and fold change >15%.

### 4.5 Targeted quantitative proteomics

Selected reaction monitoring (SRM) mass spectrometry was used to quantify protein abundance as described before (Kinter et al., 2012). Bovine serum albumin (8 pmol) was added to 60 μg (as total protein) of WAT tissue homogenate as an internal standard.

### 4.6 Rotarod

Rotarod performance was measured as described by previously using Rotamex 4/8 (Columbus Instruments, Columbus, OH, USA) (Muller et al., 2006).

### 4.7 Grip strength

Hindlimb muscle strength was determined by measuring peak force using the Digital Grip Strength meter equipped with a Hind Limb Pull Bar Assembly (Columbus Instruments, Columbus, OH, USA) essentially as described before (Zhang et al., 2014). The grip force was observed over 10 trials and the maximum force was recorded.

### 4.8 Activity and sleep

Spontaneous activity and sleep were measured as previously described (Fischer et al., 2016). Activity is measured in the number of beam breaks. Sleep is measured using a method validated by Pack et al. (2007). A sleep bout is any period of inactivity (no beam breaks) greater than or equal to 40 s. Sleep fragmentation is characterized by measuring the number of sleep bouts per hour of sleep. Data were collected during the light and dark phase over the testing period following acclimation (24‐hr, one light and one dark phase) (Pack et al., 2007).

### 4.9 Statistical analyses

In general, a two‐Way ANOVA with Tukey's post hoc test was performed using graphpad prism version 7.0b (GraphPad Software, La Jolla California). Specific statistical analyses for individual datasets are included in the Figure Legends. Kaplan–Meier estimation, Gompertz parametric modeling, log‐rank testing for differences between survival curves, and Boschloo testing for difference in extreme survival were performed with specific functions implemented in the r packages survival, eha and Exact. Quantile regression implemented in the r package quantreg was used to estimate and compare mean, median, and extreme quartile survival times for each group.

## CONFLICT OF INTEREST

None declared.

## AUTHOR CONTRIBUTIONS

S.S.D. analyzed data, prepared figures, and wrote manuscript; G.P. analyzed proteomic data, helped with statistics, and edited the manuscript; M.K. performed targeted quantitative proteomic analysis; V.D. performed lifespan studies and QMR data collection; W.C.F. generated heatmap and performed pathway analysis; K.R. performed real‐time qPCR and COX enzyme assays; D.P. did genotyping of mice and western blots; S.H. assisted with various assays; K.F. and V.S. ran and analyzed healthspan measures; C.G and J.D.W. performed lifespan data analysis; C.V. provided *Surf1* ^−/−^ mice and gave critical comments for the manuscript; A.R. gave critical suggestions for the manuscript; and H.V.R. designed the experiments, wrote and edited manuscript.

## References

[acel12769-bib-0001] Bai, Y. , Shakeley, R. M. , & Attardi, G. (2000). Tight control of respiration by NADH dehydrogenase ND5 subunit gene expression in mouse mitochondria. Molecular and Cellular Biology, 20, 805–815. 10.1128/MCB.20.3.805-815.2000 10629037 PMC85197

[acel12769-bib-0002] Baker, M. J. , Tatsuta, T. , & Langer, T. (2011). Quality control of mitochondrial proteostasis. Cold Spring Harbor Perspectives in Biology, 3(7), pii: a007559. 10.1101/cshperspect.a007559 PMC3119916 21628427

[acel12769-bib-0003] Bokov, A. F. , Garg, N. , Ikeno, Y. , Thakur, S. , Musi, N. , DeFronzo, R. A. , … Richardson, A. (2011). Does reduced IGF‐1R signaling in Igf1r+/‐ mice alter aging? PLoS ONE, 6(11), e26891 10.1371/journal.pone.0026891 22132081 PMC3223158

[acel12769-bib-0004] Copeland, J. M. , Cho, J. , Lo, T. Jr , Hur, J. H. , Bahadorani, S. , Arabyan, T. , … Walker, D. W. (2009). Extension of Drosophila life span by RNAi of the mitochondrial respiratory chain. Current Biology, 19(19), 1591–1598. 10.1016/j.cub.2009.08.016. 19747824

[acel12769-bib-0005] Deepa, S. S. , Pulliam, D. , Hill, S. , Shi, Y. , Walsh, M. E. , Salmon, A. , … Van Remmen, H. (2013). Improved insulin sensitivity associated with reduced mitochondrial complex IV assembly and activity. Federation of American Societies for Experimental Biology Journal, 27(4), 1371–1380. 10.1096/fj.12-221879. 23241310

[acel12769-bib-0006] Dell'agnello, C. , Leo, S. , Agostino, A. , Szabadkai, G. , Tiveron, C. , Zulian, A. , … Zeviani, M. (2007). Increased longevity and refractoriness to Ca(2 + )‐dependent neurodegeneration in Surf1 knockout mice. Human Molecular Genetics., 16(4), 431–444. 10.1093/hmg/ddl477. 17210671

[acel12769-bib-0007] Dillin, A. , Hsu, A. L. , Arantes‐Oliveira, N. , Lehrer‐Graiwer, J. , Hsin, H. , Fraser, A. G. , … Kenyon, C. (2002). Rates of behavior and aging specified by mitochondrial function during development. Science, 298(5602), 2398–42301. 10.1126/science.1077780. 12471266

[acel12769-bib-0009] Fischer, K. E. , Gelfond, J. A. , Soto, V. Y. , Han, C. , Someya, S. , Richardson, A. , & Austad, S. N. (2015). Health effects of long‐term rapamycin treatment: The impact on mouse health of enteric rapamycin treatment from four months of age throughout life. PLoS ONE, 10(5), e0126644 10.1371/journal.pone.0126644. 25978367 PMC4433347

[acel12769-bib-0010] Fischer, K. E. , Hoffman, J. M. , Sloane, L. B. , Gelfond, J. A. , Soto, V. Y. , Richardson, A. G. , & Austad, S. N. (2016). A cross‐sectional study of male and female C57BL/6Nia mice suggests lifespan and healthspan are not necessarily correlated. Aging (Albany NY), 8(10), 2370–2391. 10.18632/aging.101059 27705904 PMC5115894

[acel12769-bib-0011] Fok, W. C. , Livi, C. , Bokov, A. , Yu, Z. , Chen, Y. , Richardson, A. , & Pérez, V. I. (2014). Short‐term rapamycin treatment in mice has few effects on the transcriptome of white adipose tissue compared to dietary restriction. Mechanisms of Ageing and Development, 140, 23–29. 10.1016/j.mad.2014.07.004. 25075714 PMC4167584

[acel12769-bib-0012] Ghazalpour, A. , Bennett, B. J. , Shih, D. , Che, N. , Orozco, L. , Pan, C. , … Lusis, A. J. (2014). Genetic regulation of mouse liver metabolite levels. Molecular Systems Biology, 10, 730 10.15252/msb.20135004 24860088 PMC4188043

[acel12769-bib-0013] Harman, D. (1972). The biologic clock: The mitochondria? Journal of the American Geriatrics Society, 20(4), 145–147. 10.1111/j.1532-5415.1972.tb00787.x 5016631

[acel12769-bib-0014] Holzenberger, M. , Dupont, J. , Ducos, B. , Leneuve, P. , Géloën, A. , Even, P. C. , … Le Bouc, Y. (2003). IGF‐1 receptor regulates lifespan and resistance to oxidative stress in mice. Nature, 421, 182–187. 10.1038/nature01298. 12483226

[acel12769-bib-0015] Hughes, B. G. , & Hekimi, S. (2011). A mild impairment of mitochondrial electron transport has sex‐specific effects on lifespan and aging in mice. PLoS ONE, 6(10), e26116 10.1371/journal.pone.0026116 22028811 PMC3189954

[acel12769-bib-0016] Kinter, C. S. , Lundie, J. M. , Patel, H. , Rindler, P. M. , Szweda, L. I. , & Kinter, M. (2012). A quantitative proteomic profile of the Nrf2‐mediated antioxidant response of macrophages to oxidized LDL determined by multiplexed selected reaction monitoring. PLoS ONE, 7(11), e50016 10.1371/journal.pone.0050016 23166812 PMC3500347

[acel12769-bib-0017] Kruse, S. E. , Watt, W. C. , Marcinek, D. J. , Kapur, R. P. , Schenkman, K. A. , & Palmiter, R. D. (2008). Mice with mitochondrial complex I deficiency develop a fatal encephalomyopathy. Cell Metabolism, 7(4), 312–320. 10.1016/j.cmet.2008.02.004 18396137 PMC2593686

[acel12769-bib-0018] Ladiges, W. , Van Remmen, H. , Strong, R. , Ikeno, Y. , Treuting, P. , Rabinovitch, P. , & Richardson, A. (2009). Lifespan extension in genetically modified mice. Aging Cell, 8(4), 346–352. 10.1111/j.1474-9726.2009.00491.x 19485964

[acel12769-bib-0019] Lapointe, J. , Stepanyan, Z. , Bigras, E. , & Hekimi, S. (2009). Reversal of the mitochondrial phenotype and slow development of oxidative biomarkers of aging in long‐lived Mclk1 + /‐ mice. Journal of Biological Chemistry, 284(30), 20364–20374. 10.1074/jbc.M109.006569 19478076 PMC2740461

[acel12769-bib-0020] Lee, S. S. , Lee, R. Y. , Fraser, A. G. , Kamath, R. S. , Ahringer, J. , & Ruvkun, G. (2003). A systematic RNAi screen identifies a critical role for mitochondria in *C. elegans* longevity. Nature Genetics, 33(1), 40–48. 12447374 10.1038/ng1056

[acel12769-bib-0021] Letellier, T. , Heinrich, R. , Malgat, M. , & Mazat, J. P. (1994). The kinetic basis of threshold effects observed in mitochondrial diseases: A systemic approach. Biochemical Journal, 302(Pt1), 171–174. 10.1042/bj3020171 8068003 PMC1137205

[acel12769-bib-0022] Liang, H. , Masoro, E. J. , Nelson, J. F. , Strong, R. , McMahan, C. A. , & Richardson, A. (2003). Genetic mouse models of extended lifespan. Experimental Gerontology, 38(11–12), 1353–1364. 10.1016/j.exger.2003.10.019 14698816

[acel12769-bib-0023] Lin, A. L. , Pulliam, D. A. , Deepa, S. S. , Halloran, J. J. , Hussong, S. A. , Burbank, R. R. , … Galvan, V. (2013). Decreased in vitro mitochondrial function is associated with enhanced brain metabolism, blood flow, and memory in Surf1‐deficient mice. Journal of Cerebral Blood Flow and Metabolism., 33(10), 1605–1611. 10.1038/jcbfm.2013.116 23838831 PMC3790931

[acel12769-bib-0024] Mansouri, A. , Muller, F. L. , Liu, Y. , Ng, R. , Faulkner, J. , Hamilton, M. , … Van Remmen, H. (2006). Alterations in mitochondrial function, hydrogen peroxide release and oxidative damage in mouse hind‐limb skeletal muscle during aging. Mechanisms of Ageing and Development, 127(3), 298–306. 10.1016/j.mad.2005.11.004 16405961

[acel12769-bib-0025] Migliaccio, E. , Giorgio, M. , Mele, S. , Pelicci, G. , Reboldi, P. , Pandolfi, P. P. , … Pelicci, P. G. (1999). The p66shc adaptor protein controls oxidative stress response and life span in mammals. Nature, 402, 309–313. 10580504 10.1038/46311

[acel12769-bib-0026] Muller, F. L. , Song, W. , Liu, Y. , Chaudhuri, A. , Pieke‐Dahl, S. , Strong, R. , … Van Remmen, H. (2006). Absence of CuZn superoxide dismutase leads to elevated oxidative stress and acceleration of age‐dependent skeletal muscle atrophy. Free Radical Biology & Medicine, 40(11), 1993–2004. 10.1016/j.freeradbiomed.2006.01.036 16716900

[acel12769-bib-0027] Munkácsy, E. , & Rea, S. L. (2014). The paradox of mitochondrial dysfunction and extended longevity. Experimental Gerontology, 56, 221–233. 10.1016/j.exger.2014.03.016 24699406 PMC4104296

[acel12769-bib-0028] Pack, A. I. , Galante, R. J. , Maislin, G. , Cater, J. , Metaxas, D. , Lu, S. , … Peters, L. L. (2007). Novel method for high‐throughput phenotyping of sleep in mice. Physiological Genomics, 28(2), 232–238. 10.1152/physiolgenomics.00139.2006 16985007

[acel12769-bib-0029] Pharaoh, G. , Pulliam, D. , Hill, S. , Sataranatarajan, K. , & Van Remmen, H. (2016). Ablation of the mitochondrial complex IV assembly protein Surf1 leads to increased expression of the UPR(MT) and increased resistance to oxidative stress in primary cultures of fibroblasts. Redox Biololgy, 8, 430–438. 10.1016/j.redox.2016.05.001 PMC4878459 27208630

[acel12769-bib-0030] Pulliam, D. A. , Deepa, S. S. , Liu, Y. , Hill, S. , Lin, A. L. , Bhattacharya, A. , … Van Remmen, H. (2014). Complex IV‐deficient Surf1(‐/‐) mice initiate mitochondrial stress responses. Biochemistry Journal, 462(2), 359–371. 10.1042/BJ20140291 PMC4145821 24911525

[acel12769-bib-0031] Ramsey, J. J. , Tran, D. , Giorgio, M. , Griffey, S. M. , Koehne, A. , Laing, S. T. , … McDonald, R. B. (2014). The influence of Shc proteins on life span in mice. The Journals of Gerontology. Series A, Biological Sciences and Medical Sciences, 69(10), 1177–1185. 10.1093/gerona/glt198 PMC4172037 24336818

[acel12769-bib-0032] Richardson, A. , Fischer, K. E. , Speakman, J. R. , de Cabo, R. , Mitchell, S. J. , Peterson, C. A. , … Austad, S. N. (2016). Measures of healthspan as indices of aging in mice‐A recommendation. The Journals of Gerontology. Series A, Biological sciences and medical sciences, 71(4), 427–430. 10.1093/gerona/glv080 PMC4834833 26297941

[acel12769-bib-0033] Rossignol, R. , Faustin, B. , Rocher, C. , Malgat, M. , Mazat, J. P. , & Letellier, T. (2003). Mitochondrial threshold effects. Biochemical Journal, 370(Pt 3), 751–762. 10.1042/bj20021594 12467494 PMC1223225

[acel12769-bib-0034] Selman, C. , Lingard, S. , Choudhury, A. I. , Batterham, R. L. , Claret, M. , Clements, M. , … Withers, D. J. (2007). Evidence for lifespan extension and delayed age‐related biomarkers in insulin receptor substrate 1 null mice. The FASEB Journal, 22, 807–818. 17928362 10.1096/fj.07-9261com

[acel12769-bib-0035] Selman, C. , Lingard, S. , Gems, D. , Partridge, L. , & Withers, D. J. (2008). Comment on “Brain IRS2 signaling coordinates life span and nutrient homeostasis”. Science, 320, 1012b 10.1126/science.1152366 18497277

[acel12769-bib-0036] Spinazzi, M. , Casarin, A. , Pertegato, V. , Salviati, L. , & Angelini, C. (2012). Assessment of mitochondrial respiratory chain enzymatic activities on tissues and cultured cells. Nature Protocols, 7(6), 1235–1246. 10.1038/nprot.2012.058 22653162

[acel12769-bib-0037] Taguchi, A. , Wartschow, L. M. , & White, M. F. (2007). Brain IRS2 signaling coordinates life span and nutrient homeostasis. Science, 317, 369–372. 10.1126/science.1142179 17641201

[acel12769-bib-0038] Tiranti, V. , Hoertnagel, K. , Carrozzo, R. , Galimberti, C. , Munaro, M. , Granatiero, M. , … Zeviani, M. (1998). Mutations of SURF‐1 in leigh disease associated with cytochrome *c* oxidase deficiency. American Journal of Human Genetics., 63(6), 1609–1621. 10.1086/302150 9837813 PMC1377632

[acel12769-bib-0039] Unnikrishnan, A. , Deepa, S. S. , Herd, H. R. , Richardson, A. (2017). Extension of lifespan in laboratory mice In Conn P. M., (Ed.), Handbook of models on human aging (2nd ed.). Chapter 19. pages 1–26. Amsterdam, The Netherlands: Elsevier (in press).

[acel12769-bib-0040] Van Remmen, H. , Ikeno, Y. , Hamilton, M. , Pahlavani, M. , Wolf, N. , Thorpe, S. R. , … Richardson, A. (2003). Life‐long reduction in MnSOD activity results in increased DNA damage and higher incidence of cancer but does not accelerate aging. Physiological Genomics, 16(1), 29–37. 10.1152/physiolgenomics.00122.2003 14679299

[acel12769-bib-0041] Vermulst, M. , Bielas, J. H. , Kujoth, G. C. , Ladiges, W. C. , Rabinovitch, P. S. , Prolla, T. A. , & Loeb, L. A. (2007). Mitochondrial point mutations do not limit the natural lifespan of mice. Nature Genetics, 39, 540–543. 10.1038/ng1988 17334366

[acel12769-bib-0042] Wang, Y. , Oxer, D. , & Hekimi, S. (2015). Mitochondrial function and lifespan of mice with controlled ubiquinone biosynthesis. Nature Communications, 6, 6393 10.1038/ncomms7393 PMC4715896 25744659

[acel12769-bib-0043] Williams, M. D. , Van Remmen, H. , Conrad, C. C. , Huang, T. T. , Epstein, C. J. , & Richardson, A. (1998). Increased oxidative damage is correlated to altered mitochondrial function in heterozygous manganese superoxide dismutase knockout mice. Journal of Biological Chemistry, 273(43), 28510–28515. 10.1074/jbc.273.43.28510 9774481

[acel12769-bib-0044] Yamate, J. , Tajima, M. , Kudow, S. , & Sannai, S. (1990). Background pathology in BDF1 mice allowed to live out their life‐span. Laboratory Animals, 24(4), 332–340. 10.1258/002367790780865976 2270043

[acel12769-bib-0045] Zhang, Y. , Bokov, A. , Gelfond, J. , Soto, V. , Ikeno, Y. , Hubbard, G. , … Fischer, K. (2014). Rapamycin extends life and health in C57BL/6 mice. The Journals of Gerontology. Series A, Biological Sciences and Medical Sciences, 69(2), 119–1130. 10.1093/gerona/glt056 PMC4038246 23682161

[acel12769-bib-0046] Zhu, Z. , Yao, J. , Johns, T. , Fu, K. , De Bie, I. , Macmillan, C. , … Shoubridge, E. A. (1998). SURF1, encoding a factor involved in the biogenesis of cytochrome c oxidase, is mutated in Leigh syndrome. Nature Genetics, 20(4), 337–343. 10.1038/3804 9843204

[acel12769-bib-0047] Zordan, M. A. , Cisotto, P. , Benna, C. , Agostino, A. , Rizzo, G. , Piccin, A. , … Costa, R. (2006). Post‐transcriptional silencing and functional characterization of the *Drosophila melanogaster* homolog of human Surf1. Genetics, 172(1), 229–241. 16172499 10.1534/genetics.105.049072 PMC1456150

