# Supplementary material for: Lifelong reduction in complex IV induces tissue‐specific metabolic effects but does not reduce lifespan or healthspan in mice
Source: Aging Cell. 2018 Apr 25;17(4):e12769. doi: 10.1111/acel.12769 (PMC6052393; doi:10.1111/acel.12769)
Supplement: Supplementary file 1 [file ACEL-17-na-s001.pptx]

## Slide 1
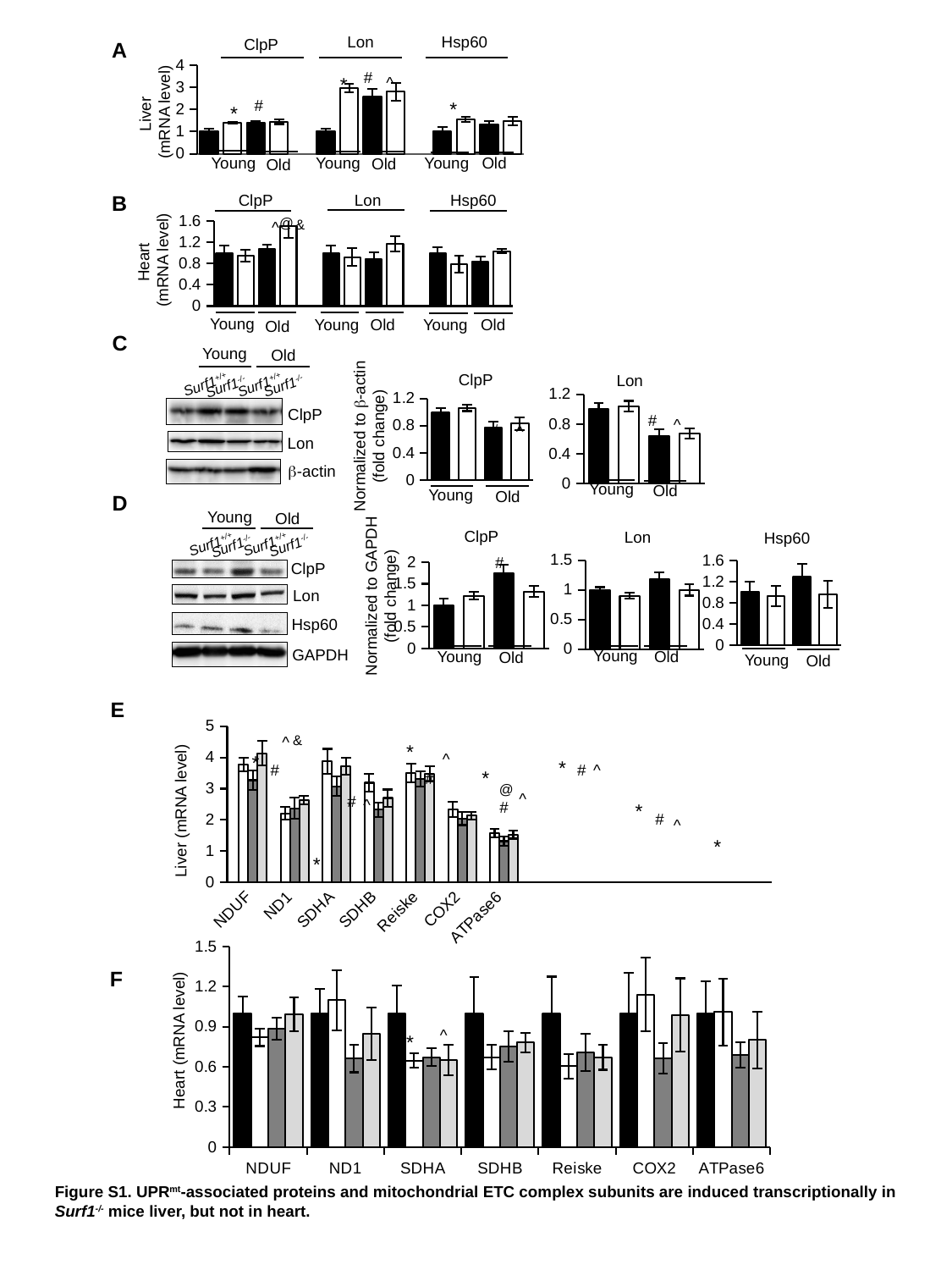

Lon
Hsp60
ClpP
A
### Chart
| Category | |
|---|---|
| WT | 1.0 |
| KO | 1.3902701951306 |
| WT | 1.400816700303306 |
| KO | 1.434470932745441 |
| | None |
| WT | 1.0 |
| KO | 2.957619874815879 |
| WT | 2.598609433315642 |
| KO | 2.796472887945967 |
| | None |
| WT | 1.0 |
| KO | 1.53995493455823 |
| WT | 1.316797281060347 |
| KO | 1.461165415193691 |#
^
*
Liver
(mRNA level)
#
*
*
Young
Young
Old
Young
Old
Old
ClpP
Lon
Hsp60
B
@
### Chart
| Category | |
|---|---|
| WT | 1.0 |
| KO | 0.948279541101558 |
| WT | 1.072072772654124 |
| KO | 1.500386030656727 |
| | None |
| WT | 1.0 |
| KO | 0.919775921680744 |
| WT | 0.88179177952333 |
| KO | 1.170946067111297 |
| | None |
| WT | 1.0 |
| KO | 0.78933528718619 |
| WT | 0.842038846335268 |
| KO | 1.0300422796146 |&
^
Heart
(mRNA level)
Young
Young
Old
Young
Old
Old
C
Young
Old
ClpP
Lon
Surf1+/+
Surf1+/+
Surf1-/-
Surf1-/-
### Chart
| Category | |
|---|---|
| WT | 1.0 |
| KO | 1.040779 |
| WT | 0.6398586 |
| KO | 0.6778362 |
### Chart
| Category | |
|---|---|
| WT | 1.0 |
| KO | 1.062013 |
| WT | 0.7678357 |
| KO | 0.8315586 |ClpP
#
^
Normalized to b-actin
(fold change)
#
^
Lon
b-actin
Young
Old
Young
Old
D
Young
Old
ClpP
Lon
Hsp60
Surf1+/+
Surf1+/+
Surf1-/-
Surf1-/-
### Chart
| Category | |
|---|---|
| WT | 1.0 |
| KO | 0.9317635 |
| WT | 1.29332 |
| KO | 0.955728 |#
### Chart
| Category | |
|---|---|
| WT | 1.0 |
| KO | 0.899807 |
| WT | 1.186667 |
| KO | 0.9996017 |
### Chart
| Category | |
|---|---|
| WT | 1.0 |
| KO | 1.220809 |
| WT | 1.738346 |
| KO | 1.316176 |ClpP
Lon
Hsp60
GAPDH
Normalized to GAPDH
(fold change)
Young
Old
Young
Old
Young
Old
E
### Chart
| Category | | | | |
|---|---|---|---|---|
| NDUF | 1.0 | 3.76476049681724 | 3.270555625473654 | 4.135501570381507 |
| ND1 | 1.0 | 2.209023508228575 | 2.375236716278707 | 2.63699826667192 |
| SDHA | 1.0 | 3.870269161544066 | 3.079183316457834 | 3.713002658824812 |
| SDHB | 1.0 | 3.188603576252591 | 2.329331126039685 | 2.701915720207696 |
| Reiske | 1.0 | 3.507926172786662 | 3.312789996313868 | 3.472150718609941 |
| COX2 | 1.0 | 2.335186552770854 | 2.042202562935501 | 2.138998659179968 |
| ATPase6 | 1.0 | 1.58059511972731 | 1.320275408185393 | 1.531870890286992 |
Liver (mRNA level)
&
^
*
^
*
*
^
#
#
*
#
@
^
#
^
#
*
#
^
*
*
### Chart
| Category | | | | |
|---|---|---|---|---|
| NDUF | 1.0 | 0.819917674806719 | 0.885969165723756 | 0.991781906285677 |
| ND1 | 1.0 | 1.099621499690086 | 0.663017512304193 | 0.845878512024877 |
| SDHA | 1.0 | 0.646954154367492 | 0.671342365580438 | 0.649657332829391 |
| SDHB | 1.0 | 0.67244125315771 | 0.752559652670522 | 0.782962873953188 |
| Reiske | 1.0 | 0.60424223651309 | 0.705161400240197 | 0.670550017173873 |
| COX2 | 1.0 | 1.141924603516642 | 0.664424809310191 | 0.988870818769099 |
| ATPase6 | 1.0 | 1.009865160111297 | 0.688838876740212 | 0.800719927906285 |Heart (mRNA level)
^
*
F
Figure S1. UPRmt-associated proteins and mitochondrial ETC complex subunits are induced transcriptionally in Surf1-/- mice liver, but not in heart.
