# Supplementary material for: Lifelong reduction in complex IV induces tissue‐specific metabolic effects but does not reduce lifespan or healthspan in mice
Source: Aging Cell. 2018 Apr 25;17(4):e12769. doi: 10.1111/acel.12769 (PMC6052393; doi:10.1111/acel.12769)
Supplement: Supplementary file 2 [file ACEL-17-na-s002.docx]

|  | **Q.4** | **Q.5** | **Q.7** | **Q.9** | **Q.95** | **pMean** |
| --- | --- | --- | --- | --- | --- | --- |
| *Surf1^+/+^* -AL vs *Surf1^-/-^*-AL | 1.8e-01 | 5.0e-02* | 0.05 * | 3.3e-01 | 7.2e-01 | 4.6e-01 |
| *Surf1^+/+^* -DR vs *Surf1^-/-^* -DR | 1.5e-02* | 5.1e-01 | 0.14 | 5.8e-01 | 7.3e-01 | 2.3e-01 |
| *Surf1^+/+^* -AL vs *Surf1^+/+^* -DR | 6.1e-12* | 1.2e-09* | 1.4e-08* | 2.2e-09* | 1.1e-02* | 7.9e-06* |
| *Surf1^+/+^* -AL vs *Surf1^-/-^* -DR | 1.1e-02* | 6.4e-04* | 2.0e-08* | 6.8e-02* | 8.6e-06* | 3.1e-03* |

**Table S1. Analysis of quartile survival time for *Surf1^+/+^* and *Surf1^-/-^* mice fed AL or DR diet.** Comparison of survival data from Figure 2 at 40% quartile of survival time (Q.4), 50% quartile (Q.5), 70% quartile (Q.7), 90% quartile (Q.9), 95% quartile (Q.95), and mean survival. * indicates significant difference between groups.

| **Liver** | | | | **Adipose** | | | | **Brain** | | | |
| --- | --- | --- | --- | --- | --- | --- | --- | --- | --- | --- | --- |
| Genes  Up | Fold chan-ge | Genes  Down | Fold change | Genes  Up | Fold cha-nge | Genes  Down | Fold change | Genes  Up | Fold cha-nge | Genes  Down | Fold change |
| HPRT1 | 3.16 | GNG2 | 2.40 | CUL3 | 3.51 | TOP2B | 16.89 | TMEM18 | 1.64 | SULF1 | 1.56 |
| CYFIP2 | 2.46 | MESDC1 | 2.32 | THBS3 | 3.33 | UBAC1 | 14.05 | FAM20B | 1.60 | SULT1A1 | 1.50 |
| CYFIP1 | 2.43 | LCMT1 | 2.13 | ADIPOQ | 3.15 | GOLGA2 | 13.66 | PFKP | 1.49 | CDIPT | 1.26 |
| GRIP1 | 2.19 | AP1S3 | 2.09 | ABTB1 | 2.93 | HINFP | 6.66 | PIGC | 1.49 | SMC6 | 1.21 |
| SEC16B | 2.18 | NSF | 1.99 | SFRP1 | 2.55 | GNS | 4.71 | TRPM8 | 1.42 | CACNA1C | 1.18 |
| ACER3 | 2.14 | CFLAR | 1.85 | GPR19 | 2.51 | RUVBL1 | 4.29 | ZCCHC7 | 1.42 | ITFG1 | 1.17 |
| USP12 | 2.12 | CDK5RAP1 | 1.82 | LNPEP | 2.51 | RXFP3 | 4.26 | PIGF | 1.39 | NFRKB | 1.17 |
| CSRP2BP | 2.04 | EHF | 1.77 | GORASP1 | 2.37 | LHFPL2 | 4.19 | ANKRD23 | 1.39 | AGRN | 1.16 |
| AOC3 | 2.03 | RABL5 | 1.75 | GRIA4 | 2.34 | L3MBTL2 | 4.06 | Etd | 1.36 | LRTM1 | 1.16 |
| MTMR6 | 2.02 | CKMT2 | 1.72 | CUL2 | 2.30 | CBS | 3.82 | Fv1 | 1.33 | FITM2 | 1.16 |

**Table S2. Tissue-specific differential regulation of pathways and genes in *Surf1^-/-^* mice.** List of genes up-regulated or down-regulated in liver, WAT, and brain of *Surf1^-/-^* mice, represented as fold change compared to *Surf1^+/+^* mice.

**
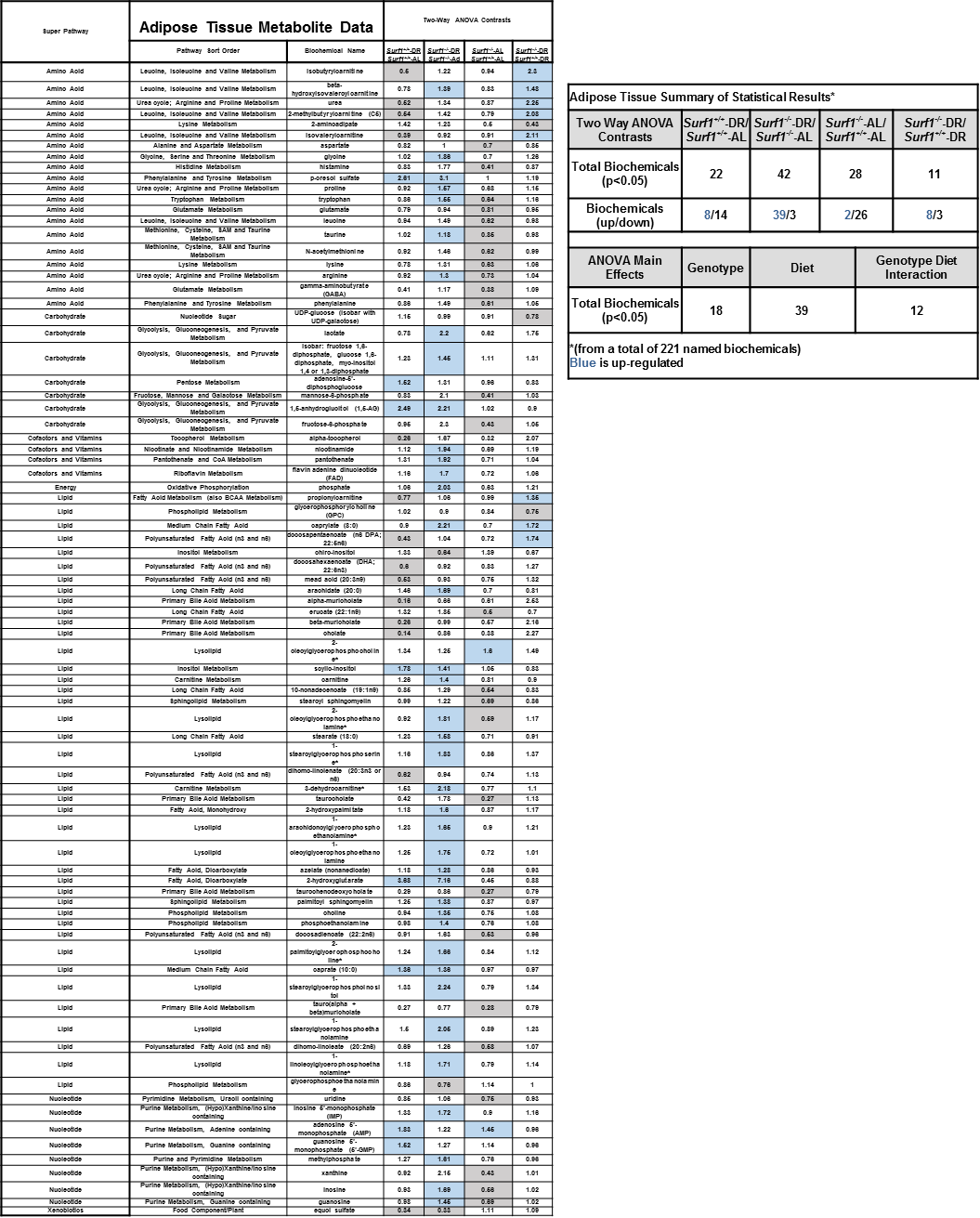
 Table S3.** **Effect of Diet and Genotype on Adipose Tissue Metabolites in *Surf1^-/-^* mice.** Left Panel: Levels of metabolites in WAT of *Surf1^+/+^* and *Surf1^-/-^* mice fed AL or DR diet. Blue color indicates significant up-regulation and grey color indicates significant downregulation. Right panel: Summary of statistical analysis.

**
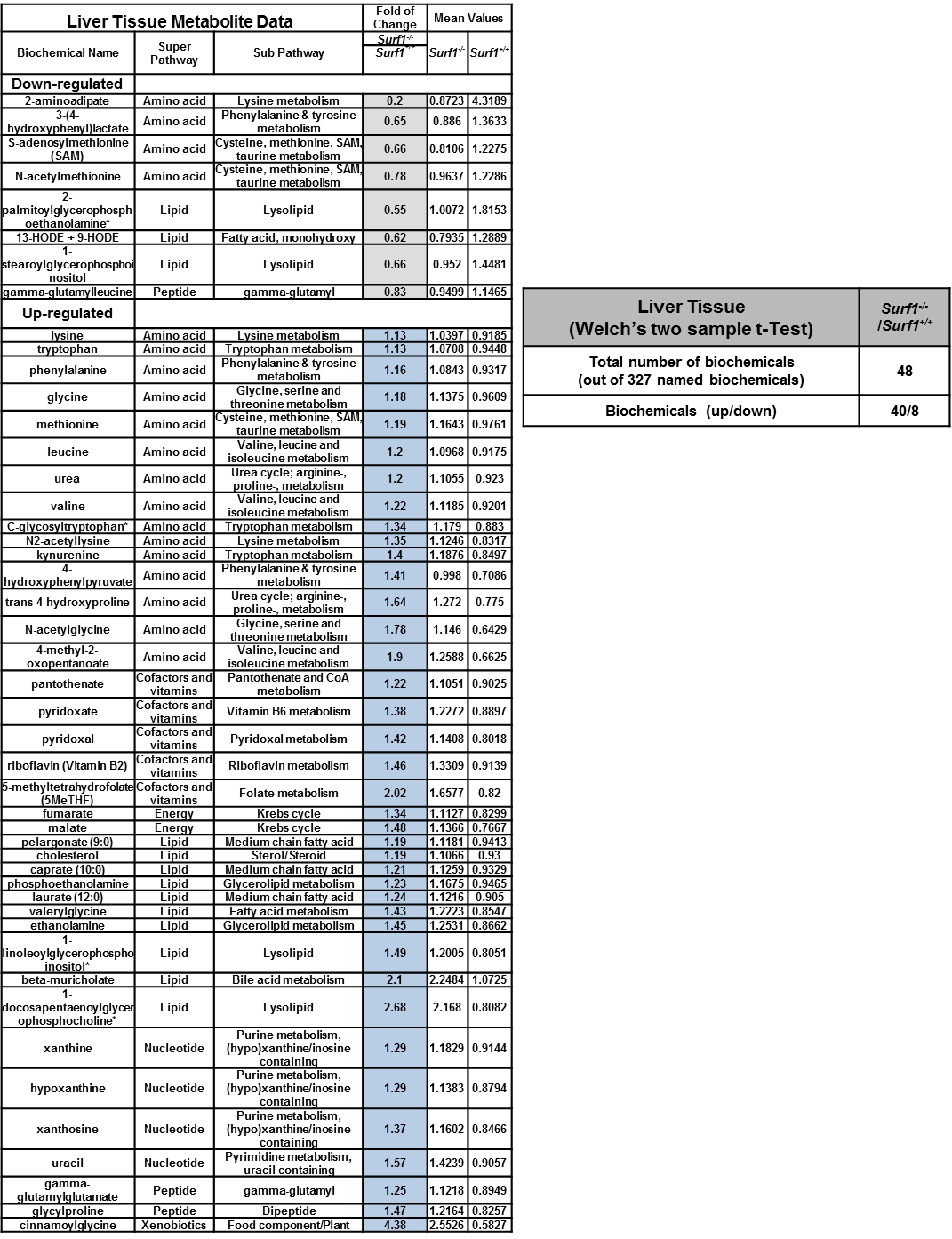
**

**Table S4.** **Changes in Liver Metabolites in *Surf1^+/+^* and *Surf1^-/-^* mice.** Left Panel: Levels of metabolites in liver of *Surf1^+/+^* and *Surf1^-/-^* mice. Blue color indicates significant up-regulation and grey color indicates significant downregulation. Right panel: Summary of statistical analysis.

**Table S5. List of proteins analyzed by targeted quantitative proteomics.** Proteins assayed by Selected Reaction Monitoring expressed as percent change relative to *Surf1^+/+^* AL. ND, not detected.
