# Supplementary material for: Lifelong reduction in complex IV induces tissue‐specific metabolic effects but does not reduce lifespan or healthspan in mice
Source: Aging Cell. 2018 Apr 25;17(4):e12769. doi: 10.1111/acel.12769 (PMC6052393; doi:10.1111/acel.12769)
Supplement: Supplementary file 3 [file ACEL-17-na-s003.docx]

**Supplementary Materials**

**Experimental Procedures**

**Western blots.** Western blot analysis was performed as we have previously described (Deepa *et al*., 2013) . Tissues were homogenized in buffer (containing 50 mM HEPES, pH 7.6; 150 mM sodium chloride; 20 mM sodium pyrophosphate; 20 mM β-glycerophosphate; 10 mM sodium fluoride; 2 mM sodium orthovanadate; 2 mM EDTA; 1.0% IGEPAL; 10% glycerol; 2 mM phenylmethylsulfonyl fluoride; and protease inhibitor cocktail. After centrifugation at 14, 000 rpm for 15 minutes at 4 ^o^C, protein content in supernatant was quantified using Bio-Rad protein assay dye reagent (Bio-Rad, Hercules, CA, USA). Prepared samples were separated by sodium dodecyl sulfate-polyacrylamide gel electrophoresis and transferred to a polyvinylidene fluoride membrane. Membrane containing bound proteins were blocked for 1 hour (1% nonfat dry milk in Tris-buffered saline/0.1% Tween 20) and probed with antibodies for ClpP (1:1000 dilution, Sigma), Lon (1:1000 dilution, gift from Dr. Luke Szweda, Oklahoma Medical Research Foundation), Hsp60 (1:1000 dilution, Cell Signaling Technology), β-actin (1:1000 dilution, Cell signaling technology) or GAPDH (1:5000 dilution, Sigma). Membranes were washed with Tris-buffered saline buffer with 0.5% Tween 20 and incubated with horseradish peroxidase-conjugated secondary antibody (Santa Cruz Biotechnology). Protein bands were visualized after adding ECL reagent using G:BOX Chemi XX6 (Syngene, Frederick, MD). Quantification of proteins were performed using GeneTools software (Syngene, Frederick, MD).

**Quantitative real-time PCR.** Total RNA was extracted from frozen liver (30 mg) and heart (30 mg) using Trizol (Ambion) according to the manufacturer’s instructions. First-strand cDNA was synthesized from 1 μg of RNA using iScript™ cDNA Synthesis Kit (Bio-Rad) as per manufacturer's instructions. Quantitative real-time PCR was performed with ABI Prism (Applied Biosystems QuantStudio® 6 Flex Real-Time PCR System) using SYBR Green PCR Master Mix (Bio-Rad) with the primers. The following primers were used for the study: ClpP (forward-GCCAAGCACACCAAACAGAG, reverse-TGGACCAGAACCTTGTCTAAGAT); Lon (forward-AGGATCTTGCCTTGTGTGGA, reverse-TGGATGAGGAGCTGAGCAAG); Hsp60 (forward-CACAGTCCTTCGCCAGATGAG, reverse-CTACACCTTGAAGCATTAAGGCT); NDUFS3 (forward-CTGTGGCAGCACGTAAGAAG, reverse-ACTCATCAAGGCAGGACACC); ND1 (forward-AATCGCCATAGCCTTCCTAAC, reverse-TGGTATTGGTAGGGGAACTCA); SDHA (forward-CAGAAGTCGATGCAGAACCA, reverse-CGACCCGCACTTTGTAATCT); SDHB (forward-GGAGGGCAAGCAACAGTATC, reverse-GCGTTCCTCTGTGAAGTCGT); Rieske (forward-TGGTCTCCCAGTTTGTTTCC, reverse-GCAGCTTCCTGGTCAATCTC); COX2 (forward-ATGGCCTACCCATTCCAACT, reverse-CGGGGTTGTTGATTTCGTC); ATPase6 (forward-ACACACCAAAAGGACGAACA, reverse-GAAGGAAGTGGGCAAGTGAG) and 18S (forward-GTGGAGCGATTTGTCTGGTT; reverse-CGCTGAGCCAGTCAGTGTAG). Calculations were performed by a comparative method (2^−ΔΔ^*^Ct^*) using 18S as control.

**Supporting Information:**

**Microarray Analysis.** RNA was extracted from liver, adipose fat, and brain using RNeasy kit (Qiagen, Valencia, CA) following manufacturer’s instructions. Biotinylated cRNA synthesis was done using Illumina TotalPrep RNA Amplification kit (Ambion, Austin, TX), hybridization to Illumina Mouse Ref8 v2.0 beadchip, and reading of microarray by Illumina iScan system was done at University of Texas Health Science Center Genomics Core. Microarray data was extracted from GenomeStudio software (Illumina, San Diego, CA) and processed through R v 3.1.0 software using Bioconductor package “lumi” (Du *et al.,* 2008). Data processed in “lumi” underwent quality control, normalized by quantile, transformed using variance stabilizing transformations, and called using a detection p-value ≤ 0.01. Statistics was done using student’s t-test in R with significance was set at p < 0.05. Significantly altered genes were visually analyzed using heatmap analysis in Matlab (2011a, The Mathworks, Natick, MA) and significantly altered genes were further analyzed by Ingenuity Pathway Analysis (Qiagen, Valencia, CA) to determine pathways that were altered. Fisher’s exact test for association of significantly changed genes into pathways and p<0.05 was then used to determine statistical significance in IPA (Du *et al.,* 2008).

**Data Deposition.** Microarray data for this study has been deposited in Gene Expression Omnibus with accession number GSE107191 and can be viewed at:   [https://urldefense.proofpoint.com/v2/url?u=https-3A__www.ncbi.nlm.nih.gov_geo_query_acc.cgi-3Facc-3DGSE107191&d=DwIFAg&c=qRnFByZajCb3ogDwk-HidsbrxD-31vTsTBEIa6TCCEk&r=oS6s5CkVDDmBIY-N4hv_3o7uyQFxUyR_0h1a6rtIeIM&m=epeW6maNeHN6X4HO9ElKWU7udwuUz-EQqeL9Mr9kOgo&s=ZO4Xgw_c9Tf96h7ADDXocp9UBXLy6km_Lg1CQrWiBKM&e=](https://webmail.ouhsc.edu/owa/redir.aspx?C=74n_ouFIY65XHw98SqwY_wi8y8TSxkkT3x0LSLlC62HTG6DgrTHVCA..&URL=https%3a%2f%2furldefense.proofpoint.com%2fv2%2furl%3fu%3dhttps-3A__www.ncbi.nlm.nih.gov_geo_query_acc.cgi-3Facc-3DGSE107191%26d%3dDwIFAg%26c%3dqRnFByZajCb3ogDwk-HidsbrxD-31vTsTBEIa6TCCEk%26r%3doS6s5CkVDDmBIY-N4hv_3o7uyQFxUyR_0h1a6rtIeIM%26m%3depeW6maNeHN6X4HO9ElKWU7udwuUz-EQqeL9Mr9kOgo%26s%3dZO4Xgw_c9Tf96h7ADDXocp9UBXLy6km_Lg1CQrWiBKM%26e%3d)  (token: mlutkkgojhmfzwr).

**Figure Legends**

**Figure S1. Expression of UPR^mt^-associated proteins and markers of mitochondrial ETC altered during aging in *Surf1^-/-^* mice.** Transcript levels of ClpP, Lon and Hsp60 in liver **(A)** and heart **(B)** of *Surf1*^+/+^ (black bar) and *Surf1^-/-^* mice (white bars) fed AL. Immunoblots showing the expression level of ClpP, Lon and Hsp60 in liver **(C)** and skeletal muscle **(D)** (left panel). β-Tubulin was used as a loading control for liver and GAPDH was the loading control for skeletal muscle. Quantification of protein levels normalized to loading control is shown in the right panel. Black bars represent *Surf1*^+/+^ mice and white bars represent *Surf1^-/-^* mice. Transcript levels of ETC subunits in liver **(E)** and heart **(F)** of young *Surf1*^+/+^ (black bar), young *Surf1^-/-^* mice (white bars), old *Surf1*^+/+^ (dark grey bar), and old *Surf1^-/-^* mice (light grey bars) fed AL. Error bars represent mean ± SEM (n=4-7). Statistical significance determined by Two-Way ANOVA with Tukey’s post-hoc test. *Young *Surf1*^+/+^ vs Young *Surf1^-/-^*, # Young *Surf1*^+/+^ vs Old *Surf1*^+/+^, ^Young *Surf1*^+/+^ vs Old *Surf1^-/-^*, @ Young *Surf1^-/-^* vs Old *Surf1*^+/+^, & Old *Surf1*^+/+^ vs Old *Surf1^-/-^*. [*/#/^/@/&p<0.05](mailto:*/#/^/@/&p<0.05).
